# Supplementary material for: Group I Paks are essential for epithelial- mesenchymal transition in an Apc-driven model of colorectal cancer
Source: Nat Commun. 2018 Aug 27;9:3473. doi: 10.1038/s41467-018-05935-6 (PMC6110733; doi:10.1038/s41467-018-05935-6)
Supplement: Supplementary file 1 — Supplementary Information [file 41467_2018_5935_MOESM1_ESM.pdf]

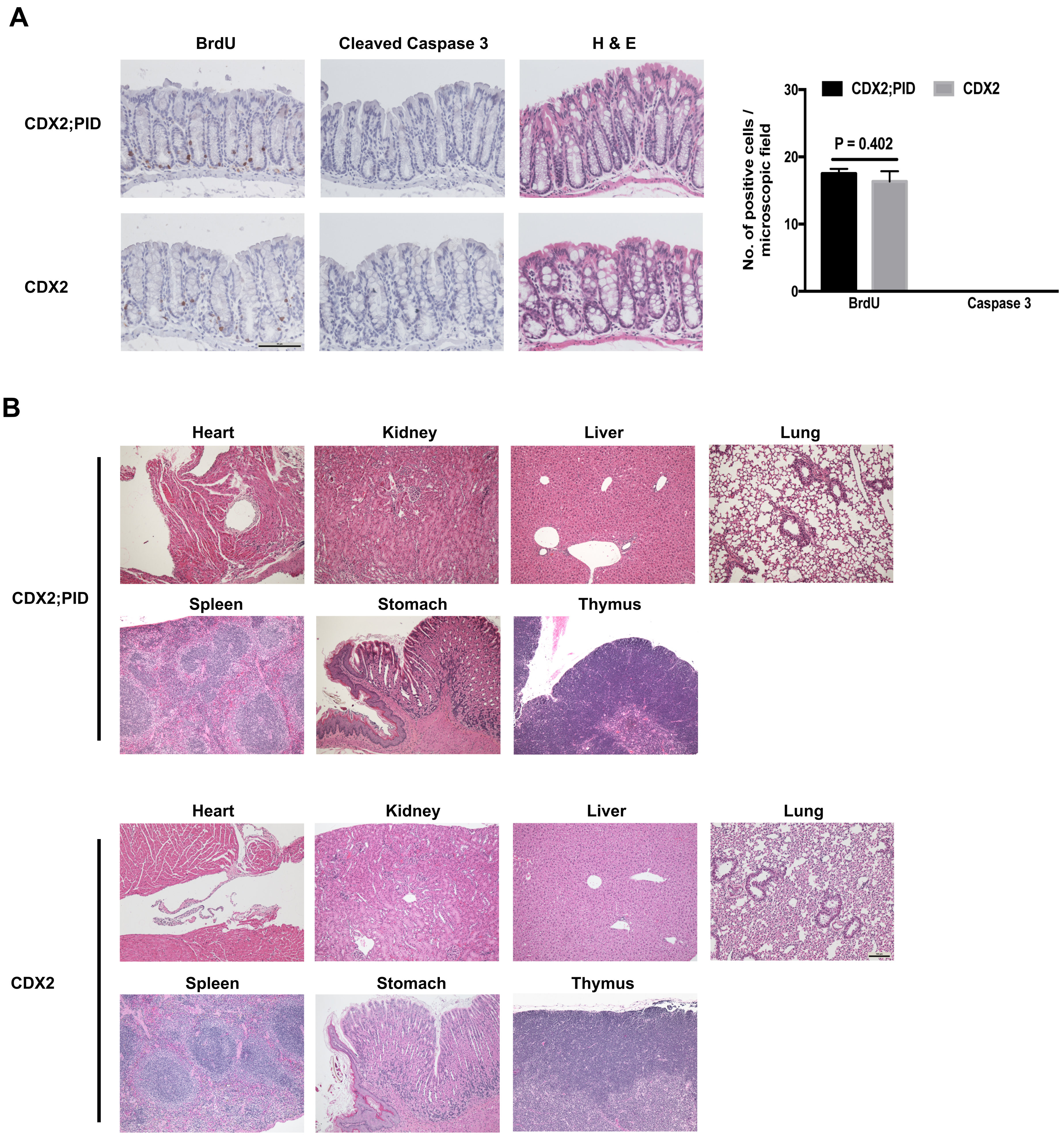

Fig. 1. Effect of PID peptide on intestinal structure of mice colon

A. Immunohistochemical staining and quantification of BrdU and Cleaved Caspase 3 in CDX2;PID and CDX2 mice colon samples, illustrating the normal cell architecture (x40 magnification). Bar, 50 μm

B. HE staining for internal organs (heart, kidney, liver, lung, spleen, stomach and thymus) in CDX2;PID and CDX2 mice (x10 magnification). Bar, 100 μm

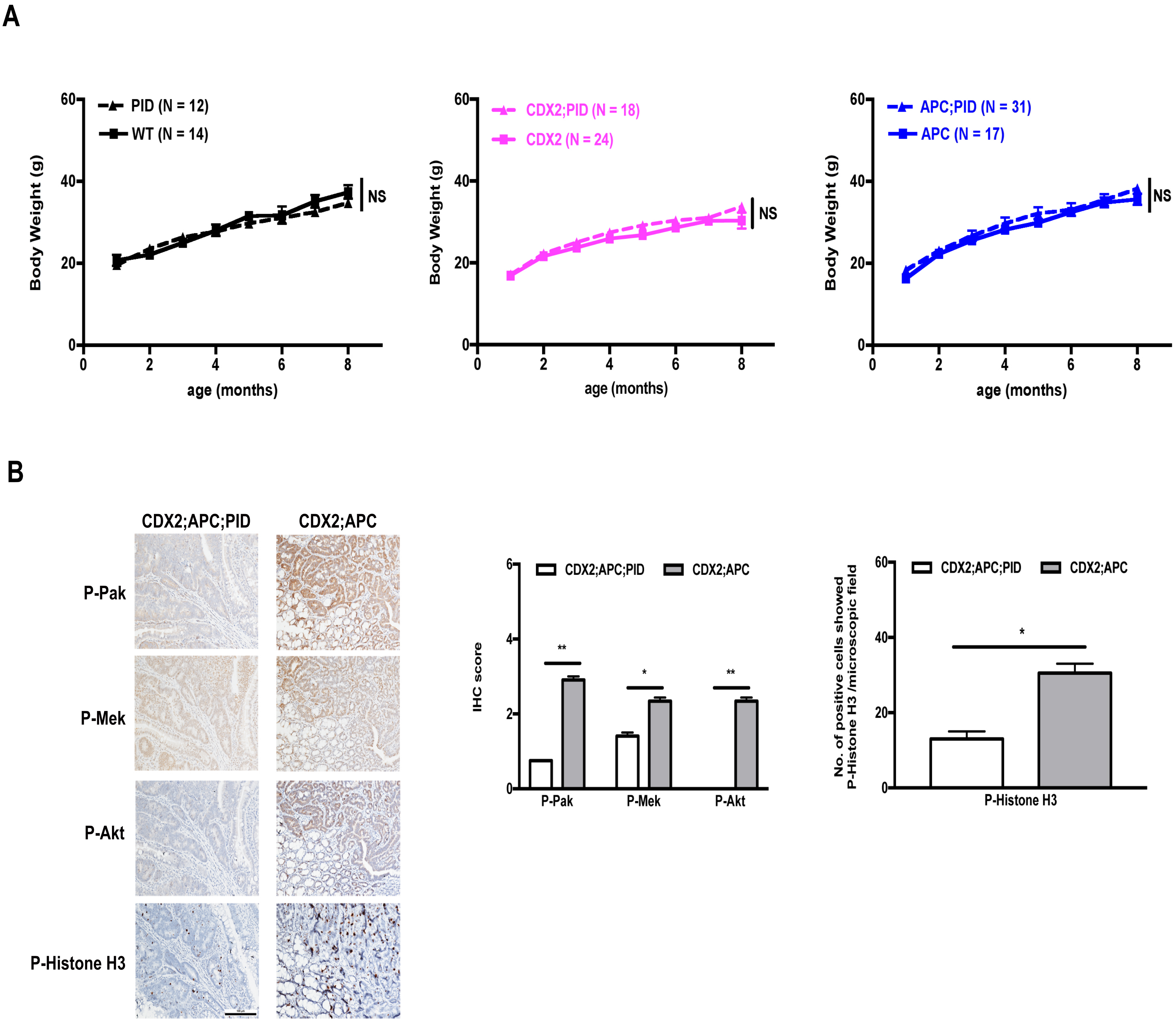

Fig. 2. Characteristics of PID expressing mice

A. Body weights of 6 groups of control mice including WT vs. PID, CDX2;PID vs. CDX2, APC;PID vs. APC. Weights were measured once every two weeks for 8 months.

B. Paraffin sections of colon tumor obtained from CDX2;APC;PID and CDX2;APC mice were subjected to immunohistological staining using antibodies against Phospho-Pak, Phospho-Mek, Phospho-Akt and Phospho-Histone H3, thus were evaluated immunoreactivity using a score from 0 to 3+ according to the intensity of stain (for p-Pak, P-Mek, and P-Akt) and number of positive cells under high-power microscopic fields (for cleaved caspase-3) (x20 magnification). Bar, 100 μm.

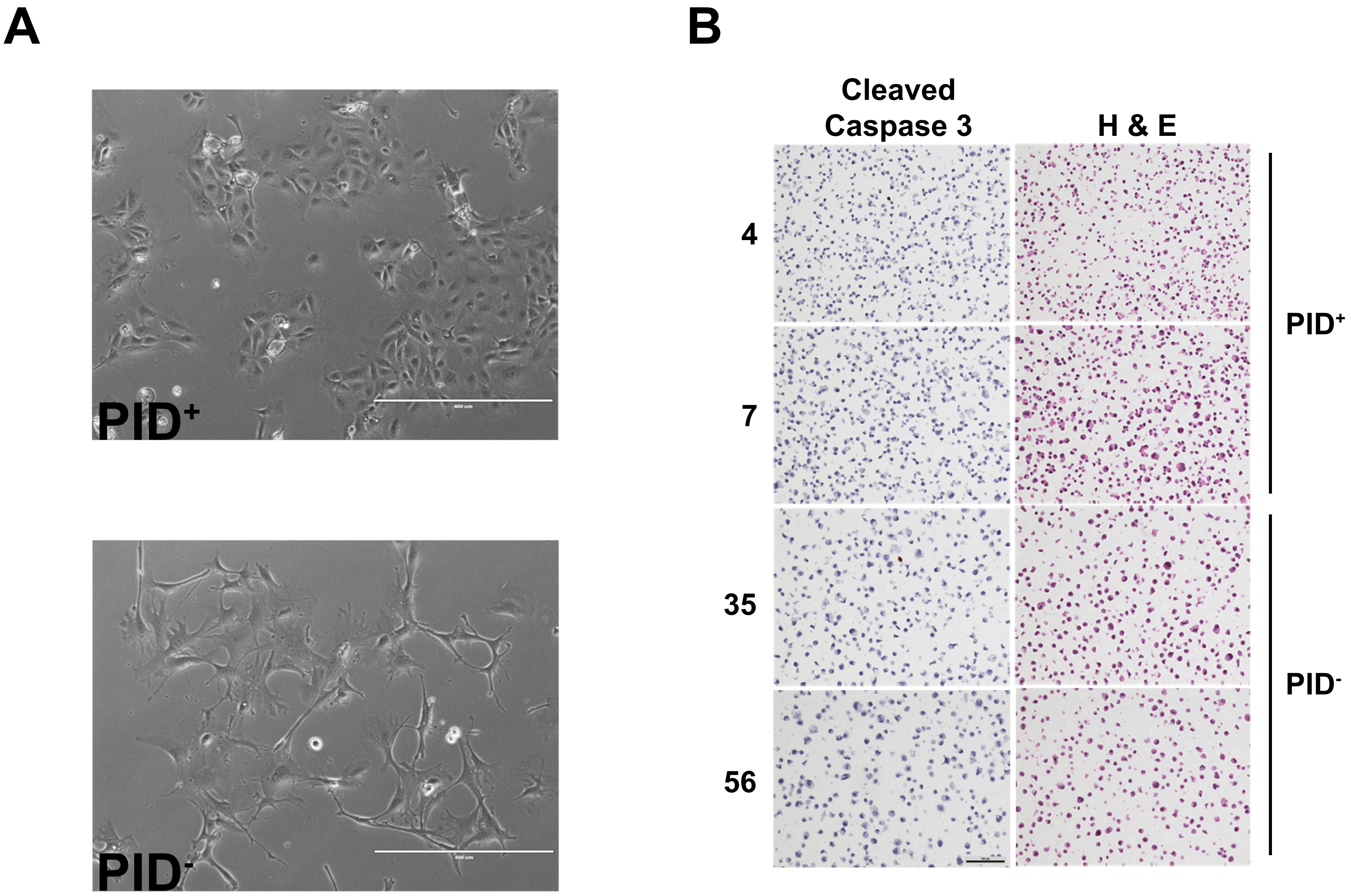

Fig. 3. Morphological change in PID- primary cells

A. The cellular morphologies of PID+ and PID- primary cells were observed under a phase-contrast microscope. Bar, 400 μm.

B. PID+ (4 and 7) and PID- (35 and 56) primary cells were fixed and embedded with HistoGel. Apoptosis was analyzed by immunohistochemistry with cleaved caspase-3. Of noted, no cells were considered positive for cleaved caspase-3 regardless of the expression of PID (x20 magnification). Bar, 100 μm.

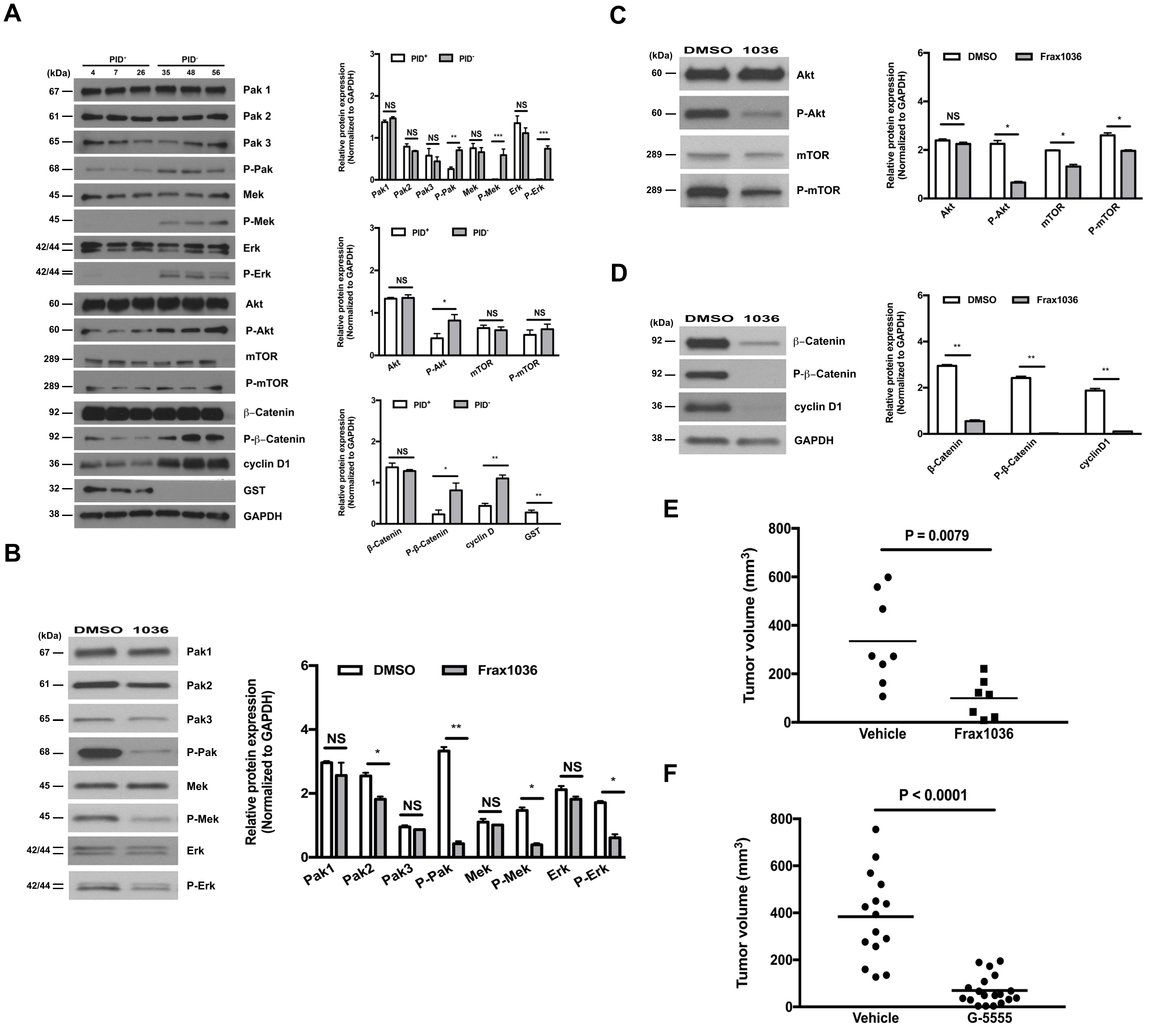

Fig. 4. Down-regulation of multiple signaling pathways in PID+ cells and the effect of Pak inhibitor on signaling pathways

A. Immunoblot analysis and quantification of MAPK and Akt-mTOR signaling pathways on cell lysates isolated from PID+ (4, 7 and 26) and PID- (35, 48 and 56) primary cells.

B. The expression levels and quantification of MAPK, Akt-mTOR (C) signaling cascade and and β-catenin (D) was assessed by immunoblot in PID- primary cells treated with Pak inhibitor, Frax-1036. GAPDH was used as a loading control.

E. Scatter plot for tumor voume in mice bearing subcutaneous SW48 tumors treated with either vehicle or FRAX-1036, once a day dose of 30 mg/kg for 2 weeks via oral gavage. Final tumor volume was determined at the end of treatment course.

F. Volumetric changes in tumor size was measured at the end of treatment course between vehicle-treated control mice and G-5555 treated littermate at dose twice of 25 mg/kg.

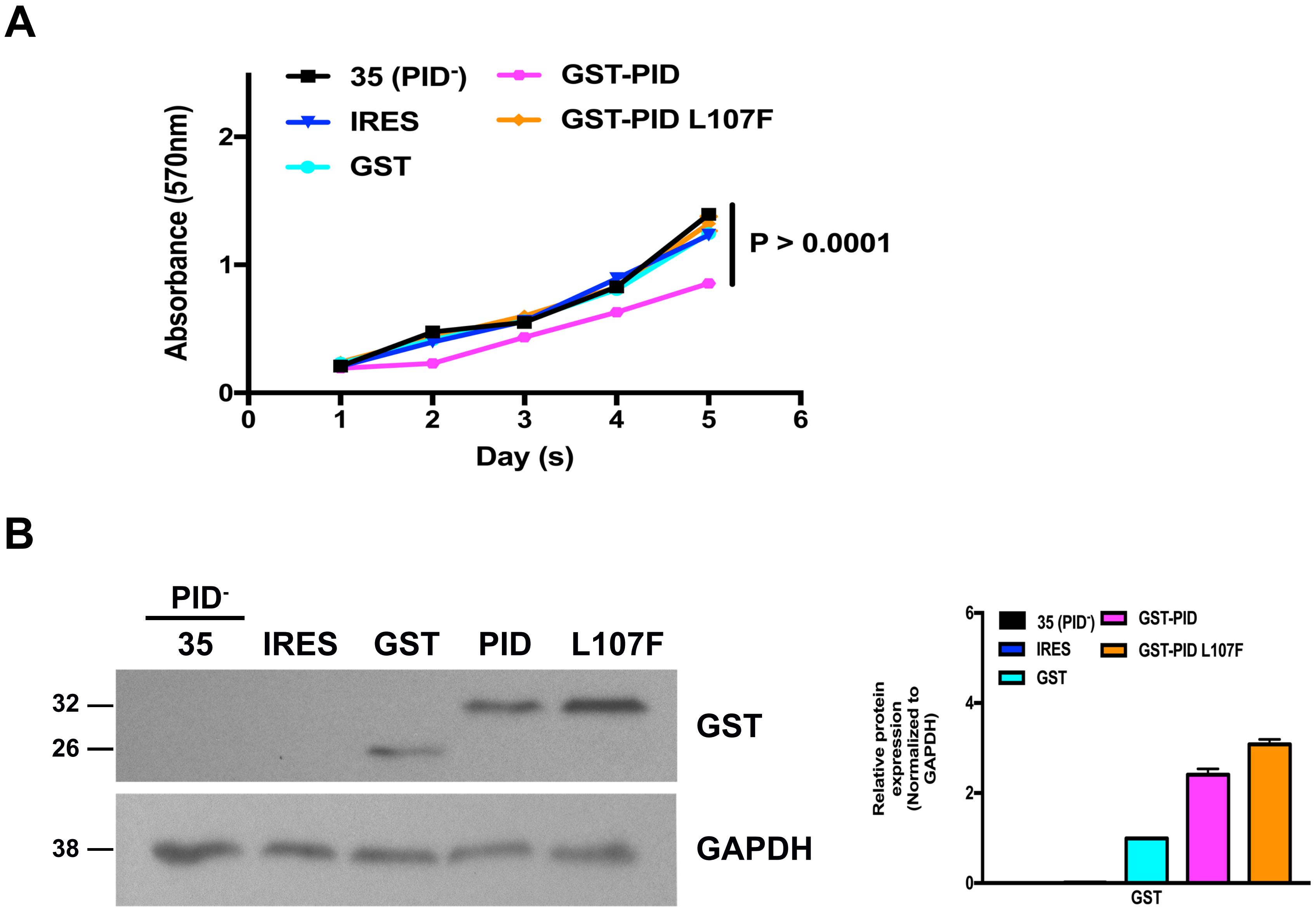

Fig. 5. Cell viability of PID- primary cells with GST constructs

A. Cell viability of PID- primary cells was performed by MTT assay, cells was transducted with retrovirus carrying empty vector (IRES), GST alone (GST), GST PID-E129K (GST-PID*), and GST-PID-E129K/L107F (GST PID* L107F), respectively.

B. Immunoblot analysis and quantification of GST on these cells described in Fig S5A.

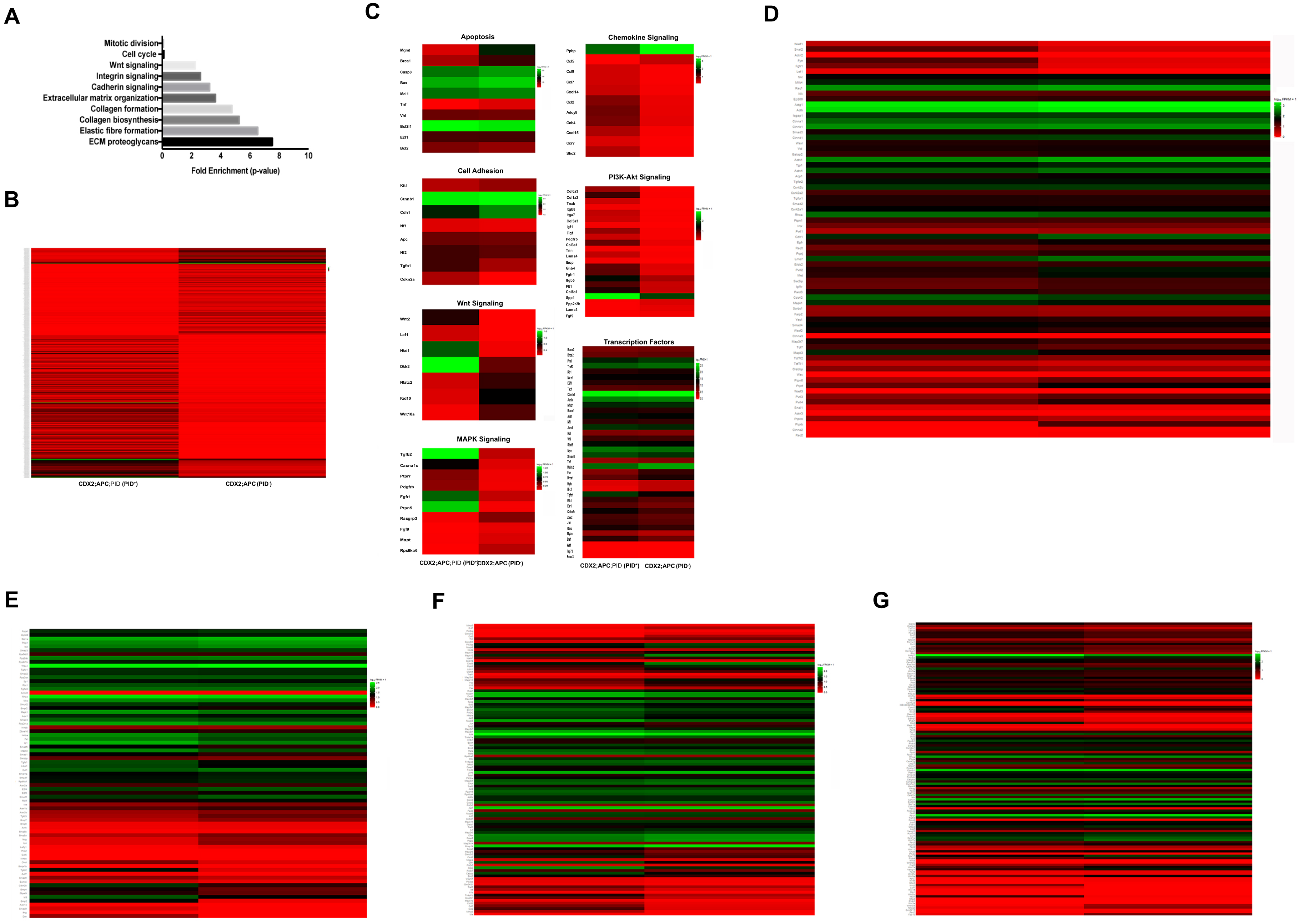

Fig. 6. Changes on transcriptome upon blocking Paks activity

A. RNA-Seq gene enrichment analysis of differentially expressed genes was performed and enriched GO biological processes categories are shown.

B. Heatmap of up- and down-regulated genes (at least 3-fold changes) in PID+ and PID- primary cells, as assessed by RNA-Seq.

C. Zoom-in images of heatmaps for some biological pathways: Apoptosis, cell adhesion, Wnt signaling, MAPK signaling, chemokine signaling, PI3K-Akt signaling, transcription factors, EMT pathway (D), TGFβ pathway (E), TNFα pathway (F), and TCF pathway (G).

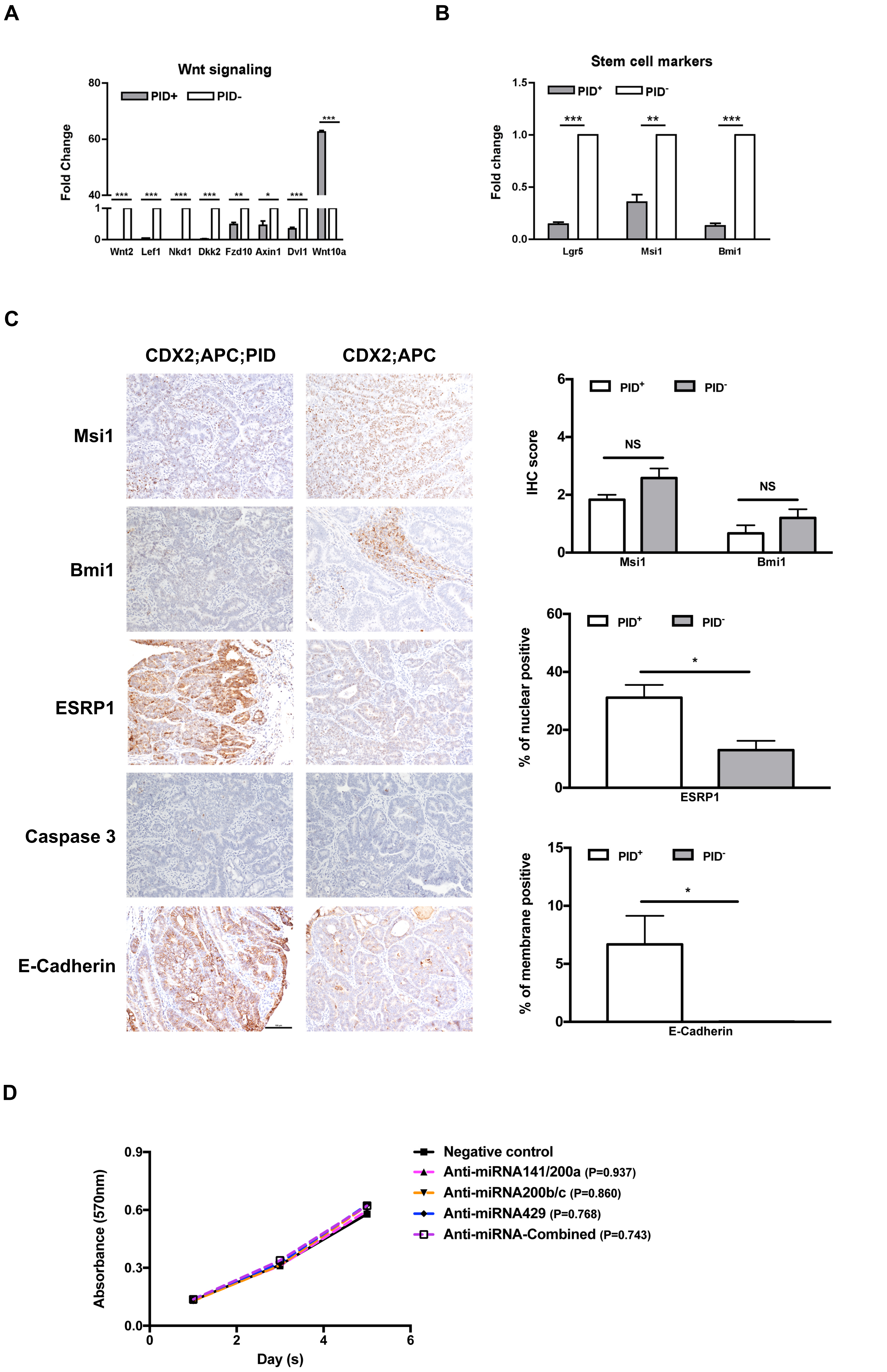

Fig. 7. Expression of stem cell and epithelial cell markers in tumor tissues

A. Relative expression level of other genes related to Wnt signaling was evaluated by real-time PCR.

B. Relative expression level of stem cell markers was evaluated by real-time PCR.

C. Paraffin sections of colon tumor obtained from CDX2;APC;PID and CDX2;APC mice were subjected to immunohistological staining using antibodies against Msi1, Bmi1, ESRP1,E-Cadherin and cleaved caspase-3, thus were evaluated immunoreactivity using a score from 0 to 3+ according to the intensity of stain (Msi1, Bmi1, ESRP1, and E-Cadherin) or , number of positive cells under high-power microscopic fields (for cleaved caspase-3) (x20 magnification). Bar, 100 μm.

D. Proliferation of PID+ primary cells transfected with either LNA-Scrambled control (Neg) or LNA-200 oligonucleotide was examined by MTT assay.

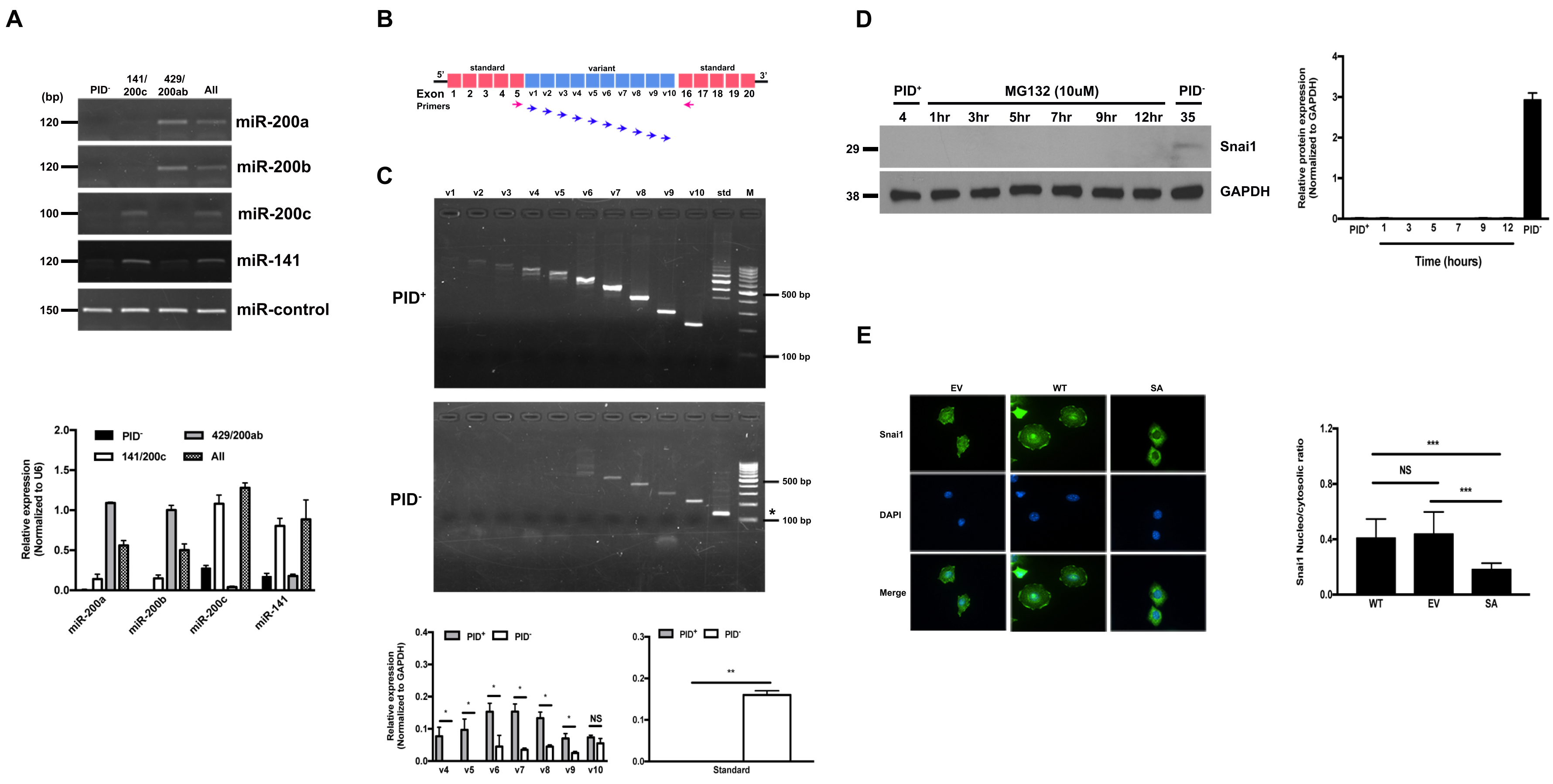

Fig. 8. Attenuated EMT by alternative splicing of CD44 and dephosphorylation and re-localization of Snai1

A. RT-PCR analysis and quantification for pre-miRNA-200 in PID- primary cells transduced with either miR-200b-200a-429 cluster or miR-200c-141 cluster. U6 served as a loading control.

B. Schematic of CD44 gene exons structure. Red boxes represent constitutive exons and blue boxes are variable exons (v1-v10) that can be alternately or entirely spliced out. Blue arrows show forward primers position for CD44 variants, red arrow located at 5' end is forward primer for standard form of CD44 whereas red arrow at 3' region is the common reverse primer for RT-PCR.

C. Exon-specific RT-PCR analysis and quantification for the expression of CD44 variants in PID+ and PID- primary cells. The lane indicated by 'std' showed PCR products obtained by using primers spanning all variant. CD44s only shown in PID- cells indicated by an asterisk. Lane 'M' showed a 100bp DNA ladder.

D. The expression level and quantification of Snai1 was assessed by immunoblot in PID+ primary cells (#4) after treatment with MG132 (10μM) for up to 12 hours. Snai1 expression in PID- cells (#35) was used as a positive control.

E. Microscopic analysis of localization of Snai1-S246A in PID- cells with anti-Snai1 antibody (Alexa-488 green) and counterstained for nuclear DNA with DAPI (blue) (x40 magnification).

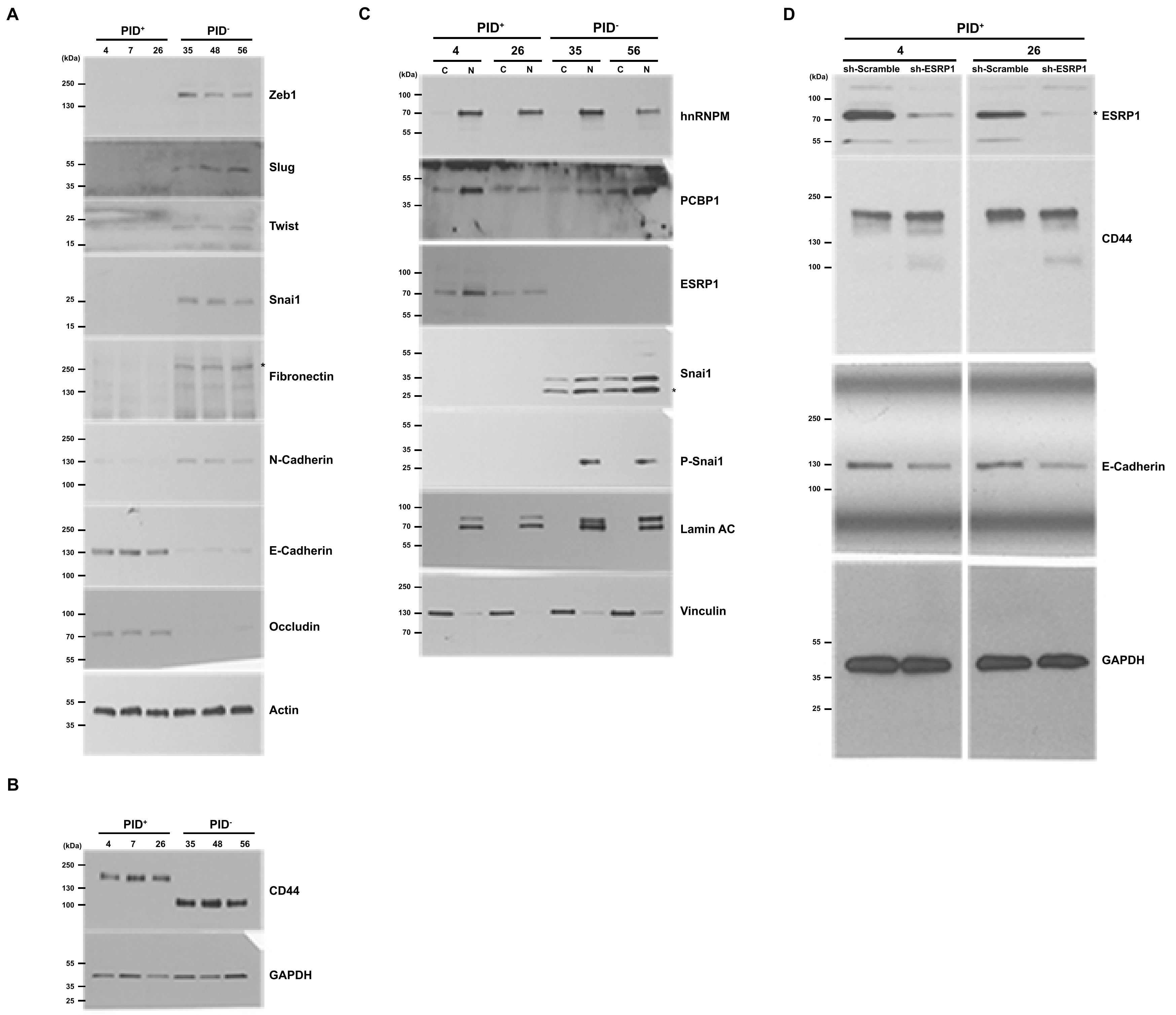

Fig. 9. Full images of selected immunoblots

A. Whole images of western blot showing expression level of EMT markers on PID+ and PID- cells in Figure 3E.

B. Whole images of western blot showing CD44 isoform expression in PID+ and PID- cells in Figure 5B.

C. Whole images of western blot showing expression and localization of hnRNPM, PCBP1, ESRP1, Snai1 and P-Snai1 in PID+ and PID- cells in Figure 5C.

D. Whole images of western blot showing expression of ESRP1, CD44, E-Cadherin and GAPDH in PID+ cells either transducted with sh-Scramble or shRNA against ESRP1 in Figure 5E.

Table 1 Real time PCR primer sequences

Gene	Forward	Reverse
**Transcription factor**		
Snai1	TCTCCTGCTCCCACTGCAAC	AGCAAGGACATGCGGGAGAA
Snai2	CACATTCGAACCCACACATT	TATTGCAGTGAGGGCAAGAG
Twist	CGGACAAGCTGAGCAAGAT	GGACCTGGTACAGGAAGTCG
Zeb1	GCTGGCAAGACAACGTGAAAG	GCCTCAGGATAAATGACGGC
Zeb2	AGATGAGCTTCCTACCGCAT	TCCCTGAAATCCTTGTTTCC
**Epithelial marker**		
CLDN	TTCCTGGATTGGTCATCAGA	AAGATGGCAGGTCCAAACTC
CRB3	CAGTGGGACTGTTTCTGCTC	ACCCACCTGCTCCTCACT
E-Caderin	GACAACGCTCCTGTCTTCAA	ACGGTGTACACAGCTTTCCA
**Mesenchymal marker**		
FN1	GGAAATTCCAATGGTGCTCT	CATCGTAGTTCTGGGTGGTG
N-Caderin	ATGCCCAAGACAAAGAAACC	CTGTGCTTGGCAAGTTGTCT
Vimentin	ATGTTAAGATGGCCCTGGAC	GGGTGTCAACCAGAGGAAGT
**Wnt signaling**		
Axin1	ACCTGCTGGACTTCTGGTTT	GTTTGTCTGGACACAATGCC
CCND1	ACCCTGACACCAATCTCCTC	GGATGGCACAATCTCCTTCT
CCND2	ATTTCAAGTGCGTGCAGAAG	AGGTAATTCATGGCCAGAGG
c-myc	TGAACCAGAGCTTCATCTGC	CTGAGAAACCGCTCCACATA
α-Catenin	TCCATGCTCTTCTGTGAAGC	ATGTCAGCCAGAATCAGCAG
β-Catenin	GCAGCAGCAGTCTTACTTGG	AGGACTTGGGAGGTGTCAAC
Dkk2	CGCTGCAATAATGGAATCTG	TTCCTAGATTCTGCCATCCC
Dvl1	GACCTGGGACTACCTCCAGA	GAACTAGCCGAGAGGAGGTG
Fzd10	CGTCTTCTCGGTGCTCTACA	CTCTTGCAGTGCTGGCTTAC

Lef1	ACACCCTGATGAAGGAAAGC	GACCCATTTGACATGTACGG
Nkd1	TGAAGAGCTGCAGTGTGATG	GGTATGCAGCAAGCTGGTAA
Wnt2	GTCCTCCTCCGAAGTAGTCG	TCTTTGGATCACAGGAGCAG
Wnt10a	GGAGACTCGGAACAAAGTCC	GCCTTCAGTTTACCCAGAGC
**Stem cell marker**		
Bmi1	ATGCTGGAGAGCTGGAAAGT	AATGTGAGGGAACTGTGGGT
Lgr5	AGGATTCCACAGCAACAACA	AAGAGAAGGGTTGCCTACGA
Msi1	TCGAAGAGCACAGCCTAAGA	TGTTTCACATCTTCCACCGT
**Matrix metalloproteinase**		
MMP2	ATCCCTGATAACCTGGATGC	TTCCAAACTTCACGCTCTTG
MMP3	GCTGAGGACTTTCCAGGTGT	CTGCGAAGATCCACTGAAGA
**CD44**		
Total	GCACTGTGACTCATGGATCC	TTCTGGAATCTGAGGTCTCC
Standard	AGAAGAGCACCCCAGAAAGC	CCTTGGATGAGTCTCGATCTC
V1	AAGCCATGCAGCAGCTCAG	
V2	ACACCACCCAAGAGGCAAG	
V3	GCTGGGAGCCAAATGAGG	
V4	TTCTGCCCGCACAGAAGAC	
V5	GACAGAATCAGCACCAGTGC	
V6	AGTACAGCAGAAGCAGCAGC	
V7	CCACAACAACCATCCAAGTC	
V8	GACTCCAGTCATAGTACAACCCT	
V9	TCTCTACATTACATGGAGAGCC	
V10	CGGCGCTAAAGATGCAAG	
**miRNA-200 family**		
141	GGGTCCATCTTCCAGTGCAGTG	GGGCCATCTTTACCAGACAGTG
200a	GCATCTTACCGGACAGTGCTG	GGGTCACCTTTGAACATCGTTACC

200b	TGGCCATCTTACTGGGCAGCA	GCCGTCATCATTACCAGGCAGTA
200c	GCAGTGTTTGGGTGCTGGTTGG	CCTCCATCATTACCCGGCAG
429	GGATGTCTTACCAGACATGG	CCGTGGATGGACGGCATTACCA
U6	CTCGCTTCGGCAGCACA	AACGCTTCACGAATTTGCGT
